# Aneurism of Palmer Arch of Left Hand

**Published:** 1891-01

**Authors:** R. Menger

**Affiliations:** San Antonio


For Daniel's Texas Medical Journal.

### ANEURISM OF PALMER ARCH OF LEFT HAND.

R. MENGER, M. D., SAN ANTONIO.

I TAKE pleasure in reporting the following interesting, and, I believe, very rare case of *aneurism*, or rather *varicose enlarged arteries* of the maine lower arteries of the left arm with aneurismatic enlarged palmer arch and hemorrhages of hand, necessitating ligation of radial artery and double ligation of ulnar artery, and followed by subsequent partial gangrene of middle hand and both left fingers, which necessitated exarticulation of part of middle hand with both fingers.

The case was under treatment of Dr. Wolff, of Marion, Texas, and the following is a short history of the case:

The subject, a stout and quite healthy looking, a hard-working German farmerwoman, aged 46 years, had, as she stated, from birth on, a pulsating tumor or tumors in left middlehand, which gave her no trouble until lately, when, from hard manual work it seems, one of the aneurysmatic sacks either inflamed or increased, and became so thin that the larger sack ruptured near the upper part of the hand, causing a very severe hemorrhage. This happened six months ago and the hemorrhage was controlled by compression. Two months ago, Aug. 30, although another hemorrhage occurred in the middle hand, and, as compression was not efficient, Dr. Wolff ligated the radial artery. On Sept. 6th, however, another severe hemorrhage occurred in the hand and also of the ulnar artery near the hand-joint, and this time the hemorrhage was such, that Dr. Wolff had to ligate the ulnaris twice at different times and at different places to control the bleeding.

On the 19th of September I was summoned by Dr. Wolff to see the case with him, as, after the last ligation, a severe swelling of the entire lower extremity occurred with later symptoms of gangrene of arm, and as amputation of the arm came into question. The entire condition of the arm during and after the late hemorrhages, Dr. Wolff said was such, that he considered amputation of the arm the only way to save her even now. Yet after careful examination and reconsidering the case, although, inasmuch as the swelling had considerably diminished since the last time Dr. Wolff had seen the patient, we concluded to only exarticulate the two meta carpal bones and secure the enlarged vessels in middlehand, for the following reason: There was yet quite good circulation in the radial artery traceable down to the smaller branches on right side of hand; the heart action was good and normal, but the pulsation of the lower extremity of left arm showed all physical signs of atheromatous degeneration. Whereas, on the left side of hand, beside the œdematous swelling of the lower arm, both fingers were mortified, the same being contracted and anæsthetic, and part of the middle hand, somewhat over midway to corpo-metacarpal joint, showing all signs of gangrene, and necessitated exarticulation.

As we were out of reach of any other assistant (about ten miles from Marion on a ranch), and the case being an urgent one, we had to operate alone, Dr. Wolff giving chloroform and ether (part aeq.) and helping ligate the vessels. In spite of compressing the upper brachial artery with the tourniquet, there was considerable hemorrhage during the exarticulation, from the immense tortuous and enlarged arteries in the hand, ten of which were ligated.

On account of some gangrenous cuticle near the finger joints, the flap on ulnar side of hand was somewhat short, but I left enough sound flap to cover most of the opposite wound. After securing all hemorrhages and careful cleaning, we dressed the stump with iodoform and iodoform cotton and put the entire arm in a well-padded splint. The woman made a splendid recovery, the flap united well, and there is no recurrance of hemorrhage.

---

The increased thirst experienced after eating salt fish is not caused by the salt it contains, as is usually believed, but certain constituents in the oil which greatly stimulates general metabolism.

